# Fluorinated Metal Phthalocyanines: Interplay between Fluorination Degree, Films Orientation, and Ammonia Sensing Properties

**DOI:** 10.3390/s18072141

**Published:** 2018-07-03

**Authors:** Darya Klyamer, Aleksandr Sukhikh, Sergey Gromilov, Pavel Krasnov, Tamara Basova

**Affiliations:** 1 Nikolaev Institute of Inorganic Chemistry SB RAS, Lavrentiev Pr. 3, Novosibirsk 630090, Russia; klyamer@niic.nsc.ru (D.K.); a_sukhikh@niic.nsc.ru (A.S.); grom@niic.nsc.ru (S.G.); 2 Department of Natural Sciences, Novosibirsk State University, 2 Pirogov street, Novosibirsk 630090, Russia; 3 Institute of Nanotechnology, Spectroscopy and Quantum Chemistry, Siberian Federal University, Krasnoyarsk 660041, Russia; kpo1980@gmail.com; 4 Reshetnev Siberian State University of Science and Technology, 82 Mira prospect, Krasnoyarsk 660049, Russia

**Keywords:** metal phthalocyanines, thin films, chemiresistive sensors, ammonia, DFT calculations

## Abstract

In this work, the sensor response of MPcF*_x_* (M = Cu, Co, Zn; *x* = 0, 4, 16) films toward gaseous NH_3_ (10–50 ppm) was studied by a chemiresistive method and compared to that of unsubstituted MPc films to reveal the effects of central metals and F-substituents on the sensing properties. A combination of atomic force microscopy and X-ray diffraction techniques have been used to elucidate the structural features of thin MPcF*_x_* films deposited by organic molecular beam deposition. It has been shown that the sensor response of MPcF_4_ films to ammonia is noticeably higher than that of MPc films, which is in good correlation with the values of binding energy between the metal phthalocyanine and NH_3_ molecules, as calculated by the density functional theory (DFT) method. At the same time, in contrast to the DFT calculations, MPcF_16_ demonstrated the lesser sensor response compared with MPcF_4_, which appeared to be connected with the different structure and morphology of their films. The ZnPcF_4_ films were shown to exhibit a sensitivity to ammonia up to concentrations as low as 0.1 ppm, and can be used for the selective detection of ammonia in the presence of some reducing gases and volatile organic compounds. Moreover, the ZnPcF_4_ films can be used for the detection of NH_3_ in the gas mixture simulating exhaled air (N_2_ 76%, O_2_ 16%, H_2_O 5%, and CO_2_ 3%).

## 1. Introduction

Ammonia is an important commercial chemical used to make fertilizers, household cleaners, and refrigerants, and is used to synthesize other chemicals. Despite its natural origin and wide distribution, ammonia is both a highly toxic and corrosive gas in its concentrated form. It is classified as an extremely hazardous substance, and is subjected to strict monitoring of its concentration in the environment, as well as in the automotive and chemical industry [1]. The detected concentration levels of ammonia depend on the application areas and can be varied in a very wide range, from ppb to hundreds ppm [1].

Recently, interest has been escalating into the study of exhaled breath as a noninvasive method of diagnostics for bronchopulmonary, cardiovascular, gastrointestinal, and other diseases [2]. Inference can be made regarding the changes in the metabolism and about the presence of a particular disease according to changes in the ratios of substances released in human breath. For example, an ammonia concentration of >1 ppm indicates renal failure in nephritis, atherosclerosis of the renal arteries, toxic affections of kidneys, and other diseases [3].

There are several ammonia detection devices described in the literature. Among those, optical gas analyzers [4,5,6], catalytic ammonia sensors [1], metal-oxide gas sensors [7,8], conducting polymer gas detectors [9,10,11], and chemiresistive sensors based on carbon nanomaterials and two-dimensional (2D) transition metal dichalcogenides [12] are used for the detection of gaseous ammonia, with their virtues and shortcomings. Electrolytic devices usually suffer from their low detection limits and limited accuracy, while optical sensors have very good sensitivity, but they are usually suited only for laboratory testing rather than for low cost portable sensors. Conducting polymer-based sensors generally suffer from irreversible sensor response and low selectivity in the presence of other gases [13].

Thin films of metal phthalocyanine (MPc) derivatives and their hybrid materials are of considerable interest as active layers of chemiresistive sensors for ammonia detection [14,15]. The introduction of various substituents into the phthalocyanine macrocycle can significantly alter the films’ structure and morphology, and in turn, leads to a change of their electrical and sensing properties [15,16]. Fluorine substituents decrease the electron density of the aromatic ring and increase the oxidation potential of the MPc molecule [17]. As a result, fluorosubstituted phthalocyanines exhibit a higher sensor response to reducing gases, such as ammonia and hydrogen [18]. The better sensor response of the ZnPcF_16_ and PdPcF_16_ films towards gaseous ammonia compared with their unsubstituted analogues was presented by Schollhorn et al. [19,20] and Klyamer et al. [15], respectively. To the best of our knowledge, only sporadic data on the structural features and sensing behavior of tetrafluorosubstituted metal phthalocyanine (MPcF_4_) films are available in the literature [15,16,21]. In our previous work [15], we studied the structure of CoPcF_4_ films deposited by thermal evaporation and their sensor response to ammonia. It has been shown that, similarly to the case of MPcF_16_, the sensor response to ammonia is noticeably higher compared with unsubstituted CoPc films. To the best of our knowledge, the systematic analysis of the interplay between the fluorination degree, films’ orientation, and sensing properties have never been carried out in the literature.

In this work, the sensor response of MPcF*_x_* (M = Cu, Co, Zn; *x* = 4, 16) films toward gaseous NH_3_ (10–50 ppm) was studied by the chemiresistive method and compared to that of unsubstituted MPc films to reveal the effects of central metals and F-substituents on the sensing properties. A combination of atomic force microscopy and X-ray diffraction techniques have been used to elucidate the structural features and molecular orientation of thin films of MPcF*_x_* deposited by organic molecular beam deposition. Density functional theory (DFT) calculations have been performed to estimate the probable structure of MPcF*_x_*-analyte complexes and their bond formation energies. The sensor characteristics of ZnPcF_4_ films were studied in more detail to demonstrate their application for the selective detection of a low concentration of ammonia (up to 0.1 ppm) in the presence of CO_2_ and water vapors, as well as in the gas mixture with the composition close to exhaled air (N_2_ 76%, O_2_ 16%, H_2_O 5%, and CO_2_ 3%).

## 2. Materials and Methods

### 2.1. Preparation and Study of Thin Films

Unsubstituted (MPc, M = Cu, Co, Zn), tetrafluorosubstituted (MPcF_4_, M = Cu, Co, Zn), and hexadecafluorosubstituted (MPcF_16_, M = Cu, Co, Zn) phthalocyanines were synthesized, according to the procedures described elsewhere [15,16], from the corresponding phthalonitrile derivatives and corresponding metal chlorides. MPcF_4_ derivatives were prepared as a statistical mixture of four regioisomers because of the various possible positions of the fluorine substituents. The isomers were not separated because of the close parameters of sublimation.

The thin films of all of the investigated phthalocyanines were deposited by an organic molecular beam deposition under a vacuum of 10^−5^ Torr, onto platinum interdigitated electrodes (Dropsens, Oviedo, Spain). The electrodes have the following dimensions: the gap between digits is 10 μm; number of digits is 125 × 2 with a digit length of 6760 μm; and cell constant is 0.0118 cm^−1^. The nominal thickness of the phthalocyanine films was about 100 nm.

XRD studies of the thin film samples were carried out using a Shimadzu XRD-7000 diffractometer (CuKα, λ = 1.54187Å, Bragg–Brentano scheme, θ-θ goniometer, copper anode sealed tube 30 mA@40 kV with a Ni filter and scintillation counter detector). The scan range was from 5° to 30° 2θ, with the step of 0.03°, and the acquisition time of 40 s per step. The atomic force microscopy (AFM) in the tapping mode with a Nanoscope IIIa (Veeco Instruments, Plainview, NY, USA) scanning probe microscope was used for the characterization of the films’ morphology.

To test the chemiresistive sensor response the films were put into the flow cell and held for 10 min under air flow until the resistance reached a steady state value. Then NH_3_ gas (0.1–50 ppm) was diluted with air and injected. Air was used as the dilution and carrier gas. The electrical resistance of phthalocyanine films was measured with a Keithley 236 electrometer by applying a constant dc voltage (8 V). All gas sensing measurements were carried out at room temperature.

### 2.2. Theoretical Calculations

The interaction of MPc, MPcF_4_, and MPcF_16_ with NH_3_ was studied by the density functional theory (DFT), using the BP86/def2-SVP method [22,23,24,25] and the Grimme D3 dispersion correction [26,27]. The ORCA suite of the quantum chemical programs was used for all of the calculations [28].

The binding energy (*E_b_*) was calculated according to the Equation (1), as a difference of the total energies of the corresponding adduct and its interacting components, as follows:

(1) $$E_{b}=E_{{\mathrm{CoPcF}}_{x}-{\mathrm{NH}}_{3}}-E_{{\mathrm{NH}}_{3}}-E_{{\mathrm{CoPcF}}_{x}}$$

The effective charge *q*(NH_3_) was calculated according to the Equation (2), as follows:

(2) $$q=\sum_{n}(Z_{n}-\sum_{I\in n}\sum_{J}P_{IJ}S_{IJ})$$

where Z_n_ is the nuclear charge of the atom n; and *P_IJ_* and *S_IJ_* are the elements of the density and overlap the matrixes corresponding to the atomic orbitals *I* and *J*. This scheme, realized in ORCA, is based on the widely used Mulliken population analysis [29,30]. A bond order was estimated using Mayer’s method [31,32].

## 3. Results and Discussion

### 3.1. Experimental Study of the Dependence of Sensing Response on Phthalocyanine Molecular Structure

**Figure 1:**
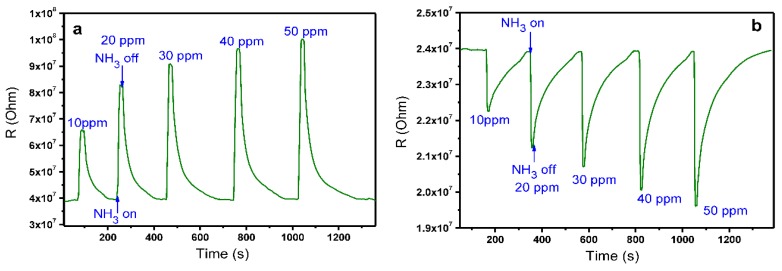
Sensor response of CoPcF_4_ (**a**) and CoPcF_16_ films (**b**) to ammonia (10–50 ppm).

The sensor response of MPcF*_x_* (M = Cu, Co, Zn; *x* = 4, 16) films was studied by a chemiresistive method. The choice of phthalocyanines of copper, cobalt, and zinc was determined by their better sensitivity to ammonia, according to the experimental data and DFT calculations performed earlier by Liang et al. [33]. The change of the film resistance during the sequential injection of the gas analyte and air purging was measured. The typical sensor response toward ammonia is shown in Figure 1, using CoPcF_4_ and CoPcF_16_ (b) films as an example.

The introduction of ammonia to the gas cell leads to the increase of resistance of the CoPc and CoPcF_4_ films. Similar behavior typical of organic semiconductor films possessing p-type conductivity [34] was also observed in the case of ZnPc, CuPc, ZnPcF_4_, and CuPcF_4_ films.

The resistance-based sensing mechanism of the semiconducting sensors has been studied in the literature [35,36]. It has been reported that the formation of the charge-transfer complexes by the coordination of O_2_ to MPc, at the air/phthalocyanine interface and at the grain boundaries, leads to the formation of oxidized MPc^+^ and O^2−^ species, and the injection of hole charge carriers into the films’ bulk [37,38]. When a p-type semiconductor gas sensor is exposed to the reducing NH_3_ gas, the electrons injected into the material through the oxidation reaction between the reducing gas and the O^2−^ species on the semiconductor surface decrease the concentration of the holes in the layer, which in turn increases the resistance of the MPc films [39].

On the contrary, the MPcF_16_ (M = Co, Cu, Zn) films exhibit a decrease of their resistance upon interaction with the electron donor NH_3_ molecules. It is known that perfluorinated metal phthalocyanines demonstrate the n-conducting behavior because of the effect of the electron-withdrawing fluorine substituents [40,41]. When an n-type semiconductor gas sensor is exposed to the reducing NH_3_ gas, ionized oxygen anions are used to oxidize the reducing gas, and the released electrons inject into the semiconducting core, which decreases the sensor resistance proportionally to the concentration of the reducing gas-analyte [36].

**Figure 2:**
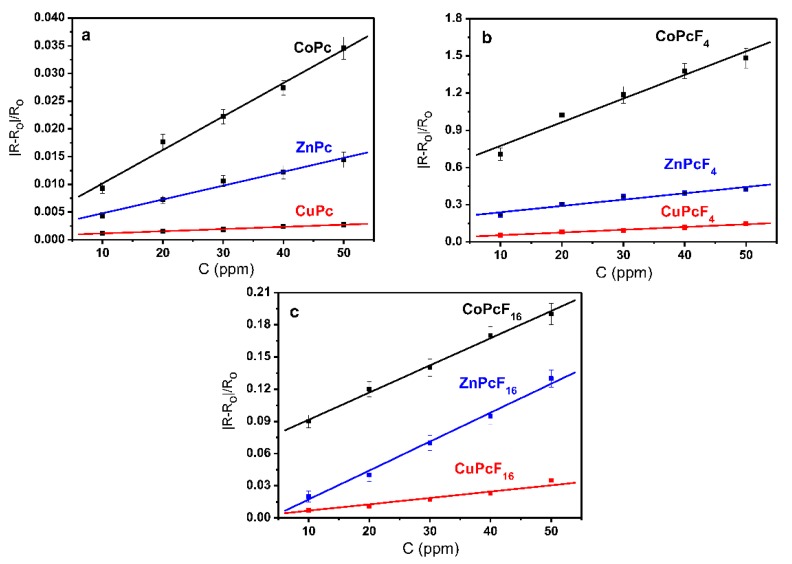
Dependence of the relative sensor response |R-R_o_|/R_o_ on NH_3_ concentration (10–50 ppm) for MPc (**a**); MPcF_4_ (**b**), and MPcF_16_ (**c**) (M = Zn, Co, Cu) films.

To study the influence of the phthalocyanine molecular structure on the sensing behavior, the sensor responses of the MPcF*_x_* (M = Co, Cu, Zn; *x* = 0, 4, 16) films toward ammonia were compared in the concentration range from 10 to 50 ppm. Figure 2 shows the dependence of the relative sensor response R_n_ = |R-R_o_|/R_o_ (where R is the resistance at a certain concentration of the analyte, R_o_ is the resistance before injection of the analyte vapors) for the MPc, MPcF_4_, and MPcF_16_ films. It can be seen that the sensor response decreases in the order of CoPcF*_x_* > ZnPcF*_x_* > CuPcF*_x_*, both in the case of the unsubstituted (Figure 2a) and fluorinated derivatives (Figure 2b,c). For instance, the sensor response of the CoPc films toward 10 ppm of ammonia is about two times higher compared with the ZnPc films, and eight times higher compared with the CuPc films (Figure 2a). An even more pronounced difference is observed in the case of the MPcF_4_ and MPcF_16_ films, for example, the sensor response of the CoPcF*_x_* (*x* = 4, 8) films toward 10 ppm of ammonia is about four times higher compared with the ZnPcF*_x_* films, and 13 times higher compared with the CuPcF*_x_* films (Figure 2b,c).

**Figure 3:**
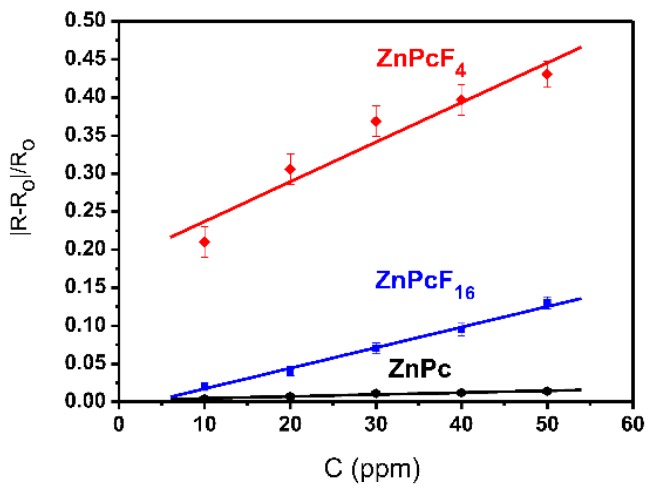
Dependence of the sensor response |R-R_o_|/R_o_ on NH_3_ concentration (10–50 ppm) for ZnPc, ZnPcF_16_, and ZnPcF_4_ films.

Figure 3 demonstrates the effect of the F-substitution in the phthalocyanine ring on the sensing response to ammonia, using the ZnPcF*_x_* (*x* = 0, 4, 16) films as an example. The sensor response decreases in the order of ZnPcF_4_ > ZnPcF_16_ > ZnPc. The same order is also observed for the CuPcF*_x_* and CoPcF*_x_* films. The MPcF_4_ films exhibit the maximal sensor response to ammonia among all of the investigated phthalocyanines, for example, the sensor response of the MPcF_4_ (M = Zn, Co, Cu) films is 3–10 times higher than that of the MPcF_16_ films, and 30–70 times higher than that of the MPc films. Therefore, the introduction of the F-substituents to the phthalocyanine macrocycle leads to a substantial increase of their sensitivity to ammonia.

**Figure 4:**
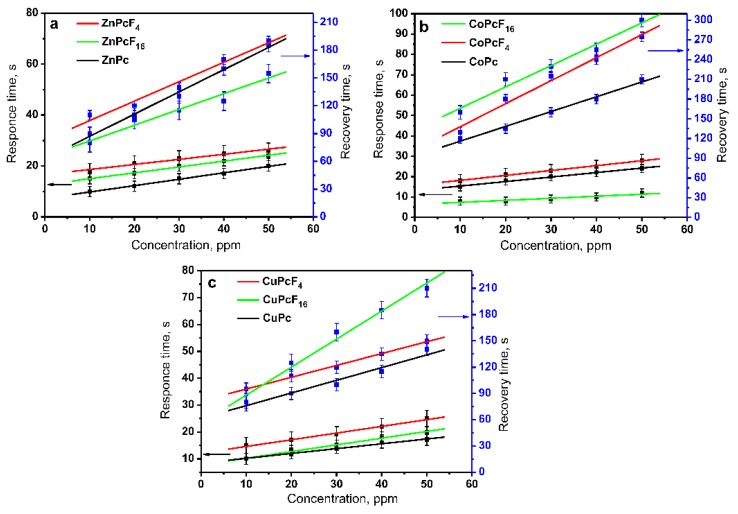
Dependence of the response and recovery times on NH_3_ concentration (10–50 ppm) for ZnPcF*_x_* (**a**), CoPcF*_x_* (**b**), and CuPcF*_x_* (**c**) (*x* = 0, 4, 16) films.

The plots of dependencies of the response and recovery times on NH_3_ concentration (10–50 ppm) for the ZnPcF*_x_* (a), CoPcF*_x_* (b), and CuPcF*_x_* (c) (*x* = 0, 4, 16) films are shown in Figure 4. The average values of the response and recovery times of all of the investigated films are also given in Table 1. All of the investigated films exhibited a reversible sensor response at room temperature, with the response time of 10–25 s. The maximal recovery times are observed in the case of the CoPcF*_x_* films, and decrease in the order of CoPcF*_x_* > ZnPcF*_x_* > CuPcF*_x_*. This order correlates with the energy of the binding of MPcF*_x_* with analyte molecules, as shown below in the Section 3.2. The more binding energy between MPcF*_x_* and NH_3_, the higher the value of recovery time is observed.

The sensor response of the sensing layers depends on several factors, among them are the molecular structure of sensing material that governs the nature and strength of its interaction with an analyte, and the sensing layer structure and morphology that determines the number of active sites, and the rate of adsorption–desorption process.

### 3.2. Theoretical Study of the Dependence of Sensor Response on the Phthalocyanine Molecular Structure

The DFT calculations have been performed to study the interaction of the NH_3_ molecules with MPcF*_x_*, and to elucidate the different sensor responses of MPcF*_x_* with different *x* and central metals. To check the validity of the theoretical model, the calculated vibrational spectra of MPcF*_x_* were compared with the experimental ones, as it has already been described elsewhere [15].

The most favorable structure of the MPc····NH_3_ aggregates simulated by the DFT calculations was that with the NH_3_ molecule binding with phthalocyanine, via its central metal. The binding of MPcF*_x_* with the NH_3_ molecule increases the out-of-plane distortion of the Pc ring (e.g., the out-of-plane displacement of the Zn atom in ZnPcF_4_ leads to an increase in the Zn-N_α_ bond length from 2.007 Å to 2.043 Å, on average). The binding parameters for NH_3_ with MPcF*_x_* are presented in Table 2 for comparison. It has already been shown elsewhere [15,42] that the ammonia and MPcs form complexes with a charge transfer from the NH_3_ to phthalocyanine molecule, via the interaction of NH_3_ with the central metal ion inside the phthalocyanine macrocycle. 

The formation of this bond is based on the displacement of electron density from NH_3_ molecule to MPc, through the central metal atom and, as a result, NH_3_ acquires a positive effective charge increasing in the order of CuPc < ZnPc < CoPc, both for the unsubstituted and fluorinated derivatives (Table 2). At the same time, the M–NH_3_ bond order increases, and the respective distance *d* between the metal atom and the ammonia nitrogen atom decreases in the same order. The obtained theoretical data are in a good correlation with the experimental investigations of the sensor response of MPc (M = Cu, Zn, Co), which is higher in the case of cobalt phthalocyanines.

As for the effect of the F-substituents, the binding energy between NH_3_ and MPcF*_x_* and the positive effective charge of NH_3_ increases in the order of MPc····NH_3_ < MPcF_4_····NH_3_ < MPcF_16_····NH_3_ (Table 2). The experimental investigations of the sensor response of the unsubstituted and fluorinated phthalocyanines showed that its value is higher in the case of MPcF_4_. However, it is necessary to mention that, in contrast to the theoretical calculations, the experimental sensor response of the MPcF_4_ films is higher than that of MPcF_16_ films. It is conceivable that such behavior can be associated with different semiconductor properties and the mechanisms of conductivity of the MPcF_4_ and MPcF_16_ films. It has already been mentioned above that the MPcF_4_ films possess the *p*-type conductivity, whereas the MPcF_16_ films demonstrate the *n*-conducting behavior. One more important factor governing the sensing properties is the structure and morphology of the sensing layers.

### 3.3. Characterization of Thin Films

**Figure 5:**
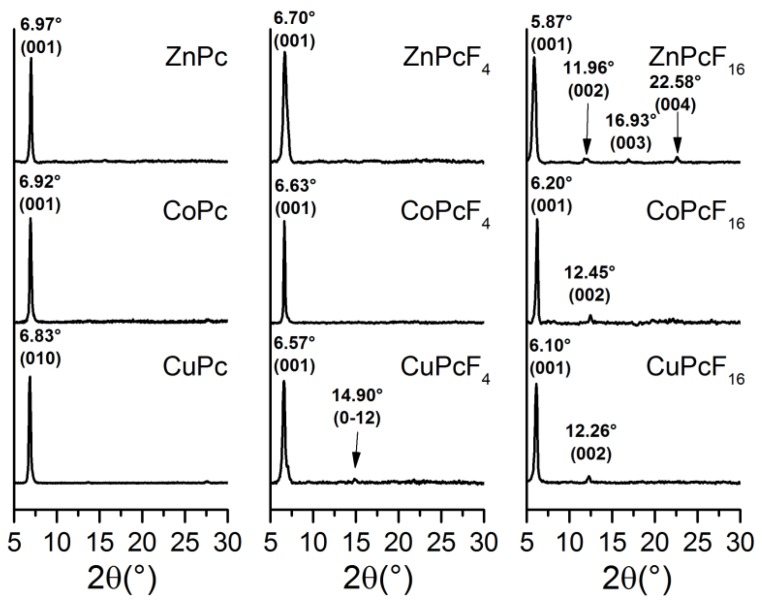
XRD patterns for thin film samples of MPcF*_x_* (M = Zn, Co, Cu; *x* = 0, 4, 16).

To study the effect of fluorination, the structure and morphology of the MPcF*_x_* films were investigated by XRD and AFM methods. X-ray diffraction patterns of thin films of all nine phthalocyanine derivatives are shown in Figure 5. The diffraction patterns contain a single strong diffraction peak in the range from 5° to 7° 2θ and several barely visible peaks with the corresponding interplanar distances *d*, which are the natural fractions of the *d*_0_ of the strong peak. This type of diffraction patterns is a typical feature of thin films with a strong preferred orientation. Comparing the interplanar distances with the calculated ones known from the single crystal data [43,44], the CoPc and CuPc thin films were identified as metastable α-polymorphs. There are no known structural data for α-ZnPc, however, some works show that α-ZnPc is isostructural to α-CuPc and α-CoPc, and it forms when deposited onto the substrate surface at temperatures lower than 100 °C [45].

CuPcF_4_, CoPcF_4_, and ZnPcF_4_ are isostructural with PdPcF_4_ [16], and crystallize only in one triclinic (P-1 space group) phase. There are two known polymorphs for CuPcF_16_, that is, α-CuPcF_16_ (P-1 space group, Z = 1) [46] and triclinic β-CuPcF_16_ (P-1 space group, Z = 2) [47], and both of them have very similar values of interplanar distances for the first peak on the calculated diffraction pattern. α-CuPcF_16_ grows on the substrate surface at room temperature, while the β-CuPcF_16_ are obtained at 360 °C. As, in this work, the substrate temperature was about 20 °C, it is reasonable to assume that the CuPcF_16_ thin films consists of a α-phase. No crystal structure data are known for the α-polymorphs of CoPcF_16_ and ZnPcF_16_, but as α-CuPc/α-CoPc, CuPcF_4_/CoPcF_4_/ZnPcF_4_, and β-CuPcF_16_/β-CoPcF_16_/β-ZnPcF_16_ are isostructural to each other, we assumed that the CoPcF_16_ and ZnPcF_16_ thin films are also α-polymorphs, with the same structure as α-CuPcF_16_.

**Figure 6:**
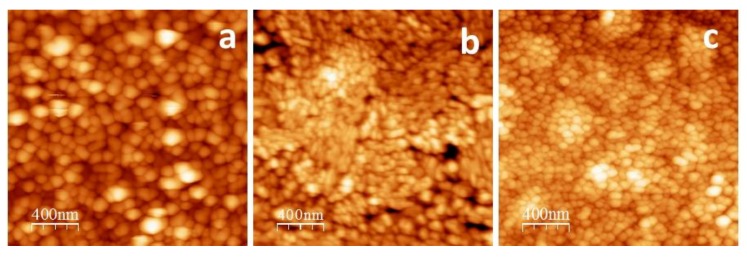
AFM images of ZnPc (**a**); ZnPcF_4_ (**b**); and ZnPcF_16_ (**c**) films.

Figure 6 shows the AFM images of the surface of the ZnPc (a), ZnPcF_4_ (b), and ZnPcF_16_ (c) films. As can be clearly seen, the ZnPc films surface consists of roundish grains (Figure 6a) and has the rms roughness value of 14.2 nm. The ZnPcF_4_ film, having an rms roughness of 6.7 nm, is formed by azimuthally disordered elongated grains (Figure 6b). The ZnPcF_16_ films exhibit a high density of azimuthally disordered roundish grains, with the size noticeably smaller than those of the ZnPc films and the minimal rms roughness values (4.2 nm) among the investigated films (Figure 6c). The more rough and inhomogeneous surface of the ZnPcF_4_ films can also be responsible for their higher sensor response to ammonia, compared with ZnPcF_16_ films.

### 3.4. Sensor Characteristics of Phthalocyanine Films

**Figure 7:**
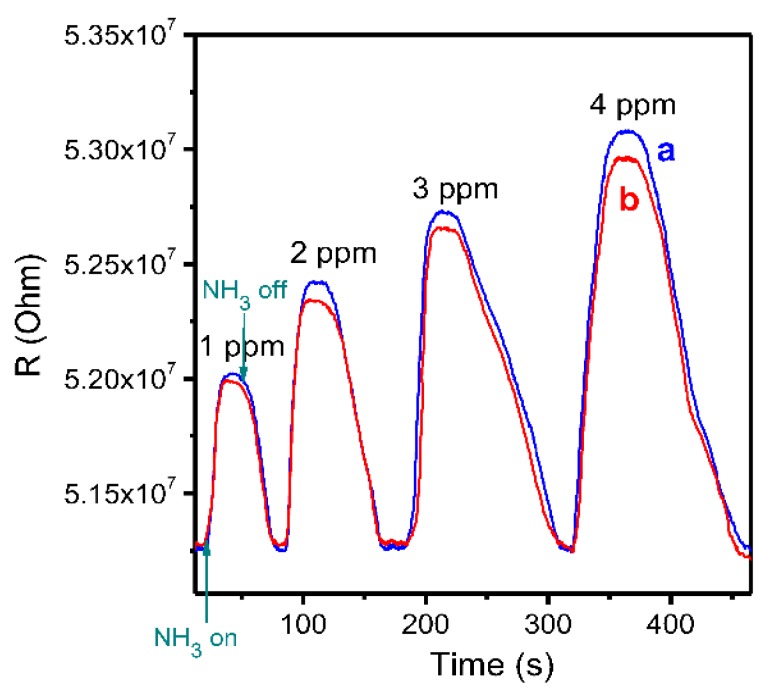
Sensor response of a ZnPcF_4_ layer toward ammonia in the concentration range from 1 to 4 ppm, in air (**a**) and in a mixture of gases with the composition close to exhaled air of healthy people (N_2_—76%, O_2_—16%, H_2_O—5%, and CO_2_—3%) (**b**).

The sensor characteristics of the MPcF_4_ films demonstrating the best sensitivity to ammonia among the investigated samples were studied in more detail to demonstrate their applicability for the detection of NH_3_ at lower concentrations, down to 0.1 ppm, in the presence of other gases. A typical sensor response of a ZnPcF_4_ layer toward ammonia, in the concentration range from 1–4 ppm, is shown in Figure 7a. To demonstrate the possible application of ZnPcF_4_ films for the detection of gases-biomarkers in exhaled air, the sensor response of ZnPcF_4_ films to ammonia was also tested in a mixture of gases, with the composition close to the exhaled air of healthy people. For this purpose, small amounts of ammonia (1–4 v.%) were added to the preliminarily prepared gas mixture (N_2_—76%, O_2_—16%, H_2_O—5%, CO_2_—3%). The sensor response of ZnPcF_4_ films to ammonia (1–4 ppm) diluted with the mixture of gases N_2_ 76%, O_2_ 16%, H_2_O 5%, and CO_2_ 3% is shown in Figure 7b.

**Figure 8:**
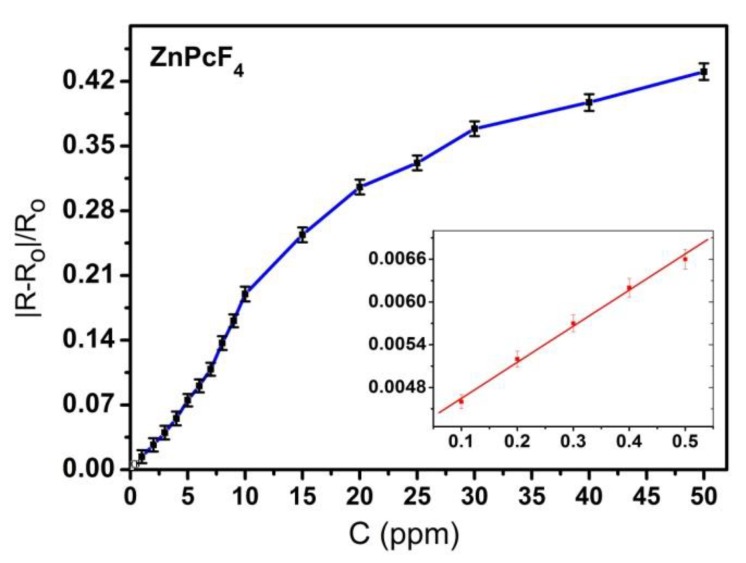
Dependence of the sensor response of ZnPcF_4_ films on NH_3_ concentration (0.1–50 ppm).

The ZnPcF_4_ films demonstrate a reversible sensor response in the investigated concentration range, with a quite good response and recovery time; the response time varied from 15 s to 30 s, depending on the NH_3_ concentration, while the recovery time increased from 28 s to 90 s when the NH_3_ concentration changes from 1 to 4 ppm. The dependence of the sensor response on the NH_3_ concentration is given in Figure 8. The minimum detected concentration of NH_3_ in the case of ZnPcF_4_ films was found to be 0.1 ppm.

**Figure 9:**
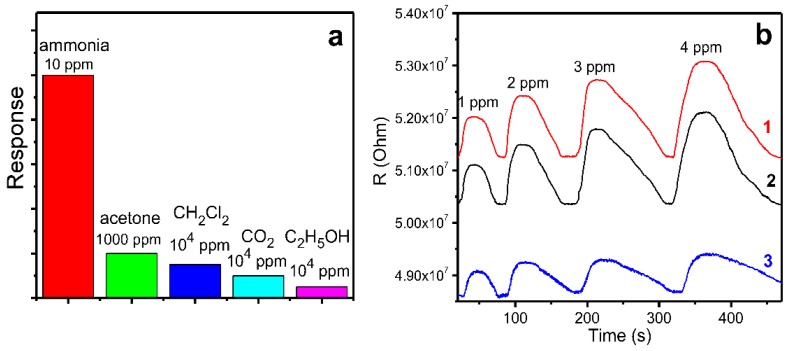
(**a**) Response of a ZnPcF_4_ film to ammonia (10 ppm), acetone (1000 ppm), dichloromethane (10^4^ ppm), carbon dioxide (10^4^ ppm), and ethanol (10^4^ ppm); (**b**) Response of a ZnPcF_4_ film to ammonia (1–4 ppm) in air measured at relative humidity of 5% (1), 30% (2), and 70% (3).

To study the selectivity of ZnPcF_4_-based sensors, their response was tested against ammonia (10 ppm), acetone (1000 ppm), dichloromethane (10^4^ ppm), carbon dioxide (10^4^ ppm), and ethanol (10^4^ ppm). Figure 9a shows that the sensor exhibited a significantly higher response to ammonia in comparison with that toward the other investigated analytes. This obviously indicates the viability of this type of sensors to detect ammonia selectively in the presence of other gases, such as those tested in this work. Note that the investigated interfering gases were taken at a much higher concentration compared with the ammonia.

The dependence of the sensor response on the relative humidity (RH) was also examined and the results are presented in Figure 9b, which show that the initial resistance of the ZnPcF_4_ films decreases with the increase of RH from 5% to 70%. The value of the sensor response to NH_3_ at RH 5% and 30% is almost the same, however, it is found to decrease noticeably when increasing the RH to 70%. The main reason for such behavior appears to be a competitive sorption of the NH_3_ and H_2_O molecules on the surface of the ZnPcF_4_ film.

The sensor response of a ZnPcF_4_ layer toward ammonia in the air was also compared with that in a mixture of gases, with the composition close to the exhaled air of healthy people. Figure 7b shows that the value of the sensor response to NH_3_ in the presence of gas mixture (N_2_ 76%, O_2_ 16%, H_2_O 5%, and CO_2_ 3%) is almost the same as in the mixture with air. This makes the ZnPcF_4_ films a promising sensing layer for the detection of ammonia in exhaled air, which is used as a gas-biomarker of renal failure in nephritis, atherosclerosis of the renal arteries, and toxic affections of the kidneys [3].

Note that the sensor performance of several sensors towards ammonia has been reported in the literature [48,49,50,51,52,53]. Some examples of sensor characteristics of several sensors, including the data obtained in this work, are summarized in Table 3 for comparison.

The sensing layers based on ZnPcF_4_ are quite competitive with the active layers, based on metal oxides, conducting polymers, and carbon-containing nanomaterials, described in the literature; the ZnPcF_4_ films exhibit a reversible sensor response at room temperature, a low detection limit, and low values of response and recovery times, compared with the other sensors.

## 4. Conclusions

In this work, unsubstituted metal phthalocyanines (MPc, M = Cu, Co, Zn), tetrafluorosubstituted metal phthalocyanines (MPcF_4_) and hexadecafluorosubstituted metal phthalocyanines (MPcF_16_) thin films were deposited by organic molecular beam deposition and studied, to reveal the effects of the central metals and F-substituents on the films’ sensor response to ammonia.

It has been shown that the sensor response decreased in the order of CoPcF*_x_* > ZnPcF*_x_* > CuPcF*_x_*, both in the case of the unsubstituted and fluorinated derivatives. The sensor response of the MPcF_4_ films to ammonia is noticeably higher than that of the MPc films, which is in good correlation with the values of the binding energy between the metal phthalocyanine and NH_3_ molecule, as calculated by the DFT method. At the same time, in contrast to the DFT calculations, MPcF_16_ demonstrated the lesser sensor response compared with MPcF_4_, which appeared to be connected with the different structure and morphology of their films.

It has been shown, using ZnPcF_4_ films as an example, that they exhibit a sensitivity to ammonia, up to concentrations as low as 0.1 ppm, and can be used for the selective detection of ammonia in the presence of some reducing gases and volatile organic compounds. Moreover, the ZnPcF_4_ films can be used for the detection of NH_3_ in the gas mixture simulating exhaled air (N_2_ 76%, O_2_ 16%, H_2_O 5%, and CO_2_ 3%). This makes these films promising active layers as chemiresistive sensors for the detection of ammonia in exhaled air, which is a biomarker of some kidney diseases.

## Figures and Tables

**Table 1:** Average values of response and recovery times of MPc, MPcF_4_, and MPcF_16_ films at the concentration of ammonia 10 ppm.

Time, s	CoPc	CoPcF_4_	CoPcF_16_	ZnPc	ZnPcF_4_	ZnPcF_16_	CuPc	CuPcF_4_	CuPcF_16_
Response	15	20	10	10	25	15	10	15	10
Recovery	120	130	160	90	110	85	80	95	80

**Table 2:** Parameters of binding of NH_3_ with MPc, MPcF_4_, and MPcF_16._

Aggregate	*E_b_*, eV	Bond Order	*d*, *Å*	*q*(NH_3_), *e*
CoPc····NH_3_	−1.14	0.484	2.153	0.243
CoPcF_4_····NH_3_	−1.16	0.486	2.152	0.245
CoPcF_16_····NH_3_	−1.20	0.491	2.151	0.250
ZnPc····NH_3_	−1.06	0.402	2.159	0.214
ZnPcF_4_····NH_3_	−1.08	0.405	2.156	0.216
ZnPcF_16_····NH_3_	−1.14	0.414	2.151	0.223
CuPc····NH_3_	−0.62	0.291	2.330	0.156
CuPcF_4_····NH_3_	−0.63	0.293	2.329	0.158
CuPcF_16_····NH_3_	−0.68	0.302	2.322	0.164

**Table 3:** Sensor performance of active layers based on metal oxides, conducting polymers, carbon-containing nanomaterials, and phthalocyanines.

Sensing Layer	Concentration Range, ppm	Minimal Investigated Concentration, ppm	Response/ Recovery Time, s	Temperature Range, °C	Ref.
**Metal Oxides**					
Pt/NiO	1–1000	0.01	15/76 (350 °C, 1000 ppm)	200–350	[7]
Pt Nanoparticle/Aluminum-Doped Zinc Oxide	1–1000	1	24/4 (350 °C, 1000 ppm)	200–350	[48]
**Conducting Polymers**					
Polyaniline/poly(styrene-butadiene-styrene)	0.1–100	0.1	≤13 (100 ppm)/--	Room temperature	[49]
Flexible polyaniline films	50–150	50	40 (50 ppm)/--	Room temperature	[50]
**Carbon-Containing Nanomaterials and Phthalocyanines**					
AuNPs/SWNT	0.25–6	0.255	20 (0.4 ppm)/--	Room temperature	[51]
rGO modified with metal tetra-α-iso-pentyloxyme-tallophthalocyanines (CuPc, NiPc, PbPc)	0.4–400	0.4	CuPc/rGO 364/115 NiPc/rGO 200/264 PbPc/rGO 248/331 (0.8 ppm)	Room temperature	[52]
CoPc on a flexible polyethylene terephthalate substrate	5–50	5	25/156 (20 ppm)	Room temperature	[53]
ZnPcF_4_	0.1–50	0.1	25/110 (10 ppm)	Room temperature	This work
